# SALSA: A Regulator of the Early Steps of Complement Activation on Mucosal Surfaces

**DOI:** 10.3389/fimmu.2016.00085

**Published:** 2016-03-08

**Authors:** Martin Parnov Reichhardt, Seppo Meri

**Affiliations:** ^1^Immunobiology Research Program, Research Programs Unit, Department of Bacteriology and Immunology, Haartman Institute, University of Helsinki, Helsinki, Finland

**Keywords:** gp340, DMBT1, Crohn’s disease, ficolins, MBL, C1q, ulcerative colitis, IBD

## Abstract

Complement is present mainly in blood. However, following mechanical damage or inflammation, serous exudates enter the mucosal surfaces. Here, the complement proteins interact with other endogenous molecules to keep microbes from entering the parenteral tissues. One of the mucosal proteins known to interact with the early complement components of both the classical and the lectin pathway is the salivary scavenger and agglutinin (SALSA). SALSA is also known as deleted in malignant brain tumors 1 and gp340. It is found both attached to the epithelium and secreted into the surrounding fluids of most mucosal surfaces. SALSA has been shown to bind directly to C1q, mannose-binding lectin, and the ficolins. Through these interactions SALSA regulates activation of the complement system. In addition, SALSA interacts with surfactant proteins A and D, secretory IgA, and lactoferrin. Ulcerative colitis and Crohn’s disease are examples of diseases, where complement activation in mucosal tissues may occur. This review describes the latest advances in our understanding of how the early complement components interact with the SALSA molecule. Furthermore, we discuss how these interactions may affect disease propagation on mucosal surfaces in immunological and inflammatory diseases.

## Introduction

Activation of the complement system is strongly involved in generating inflammation, combatting microbial infections, and participating in clearance of non-viable tissue. Although complement is present mainly in blood, it is also found in serous exudates on mucosal surfaces, such as in the oral cavity or the airways (1, 2). This is particularly seen after mechanical, infectious, or immune damage, e.g., in periodontal disease or SLE (3). When serous exudates enter the mucosal surfaces, innate immune proteins interact with mucosal surface proteins. Together, these molecules participate in clearance and defense against invading microorganisms. Although bleeding at the mucosal surfaces is observed daily, even in healthy individuals, the role of the complement system in this environment has so far been studied very little. Of particular interest would be the need to suppress complement-mediated inflammation, while still mediating the antimicrobial defense functions.

### Salivary Scavenger and Agglutinin

One of the molecules at the mucosal surfaces that interact with the early complement components is a protein that we named salivary scavenger and agglutinin (SALSA) (4–7). SALSA, also known as gp340, “deleted in malignant brain tumors 1” (DMBT1), and salivary agglutinin (SAG), was first described as a 300–400 kDa streptococcus agglutinating agent from saliva (8–10). Subsequently, SALSA has been suggested to function in epithelial homeostasis, innate immunity, inflammation, and tumor suppression (11–13). Many of these functions are mediated through interactions with endogenous ligands. SALSA has been shown to bind the complement components C1q, mannose-binding lectin (MBL), and the ficolins (4, 6). Furthermore, SALSA has been found to interact with surfactant proteins A and D (SpA and SpD, respectively), secretory IgA, lactoferrin, fibrin/fibrinogen, trefoil factors, and mucin-5B (Table 1) (9, 14–19). The multiple binding partners suggest that SALSA plays a central role in regulating inflammation and immune responses on mucosal surfaces.

**Table 1:** **Endogenous and microbial ligands of SALSA**.

Endogenous ligand	Suggested functional relevance
C1q	Complement regulation (4)
MBL	Complement regulation (6)
Ficolins	Complement regulation (6)
SpD	Microbial agglutination (9)
SpA	Microbial agglutination (16)
IgA	Microbial agglutination (8)
Lactoferrin	Bacterial binding (20)
DNA	Inflammation (21)
Heparan sulfate	Inflammation (21)
Trefoil factors	Tissue homeostasis (17)
MUC5B	Microbial agglutination (22)
Fibrin	Not known (19)
Fibrinogen	Not known (19)
Erythrocytes	Aggregation (19)
Platelets	Aggregation (19)

**Microbe**	**Specific strains**

*Streptococcus*	*S. pyogenes*, *S. agalactiae*, *S. pneumonia*, *S. mutans*, *S. mitis*, *S. oralis*, *S. salivarius*, *S. gordonii*, *S. crista*, *S. parasanguinis*, *S. vestibularis*, *S. intermedius*, *S. anginosus*, *S. suis* (7, 23–25)
*Lactobacillus*	*L. rhamnosus*, *L. casei*, *L. reuteri*, *L. lactis* (26)
Other bacteria	*Staphylococcus aureus*, *Bifidobacterium*, *Actinomyces*, *Salmonella enterica* serovar Typhimurium, *Helicobacter pylori*, *Haemophilus influenzae*, *Klebsiella oxytoca* (23–28)
Viruses	HIV, IAV (29, 30)

*The listed ligands have been found to bind either human SALSA, the murine-ortholog of SALSA, or the recombinantly expressed bacterial-binding peptide, SRCRP2*.

### SALSA in Antimicrobial Defense

Salivary scavenger and agglutinin is expressed at most mucosal surfaces, including the lungs, oral cavity, gastrointestinal tract, and vagina (31–35). It has been found both attached to the epithelium and secreted into the lining fluids, such as saliva, tear fluid, and respiratory mucosal secretions (8, 9, 14, 36). Recent studies detected SALSA in the amniotic fluid and in the intestines of neonates (37). SALSA was estimated to constitute up to 10% of the total protein amount in meconium and in the saliva of young children (<3 years), making it one of the most abundant proteins in these environments (37, 38).

**Figure 1:**
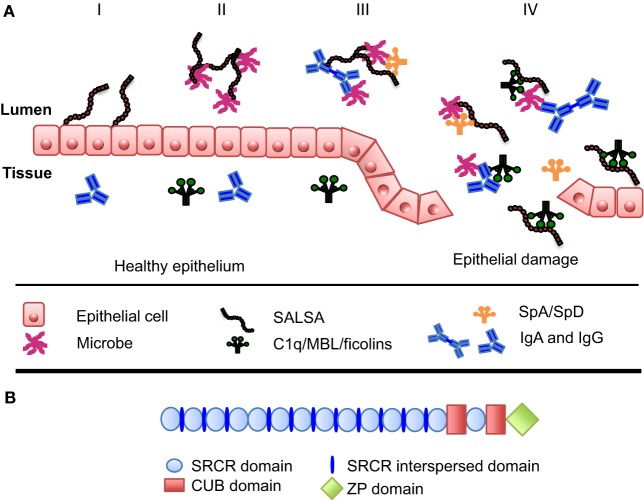
**Function and structure of SALSA at the mucosal surfaces**. **(A)** At the mucosal surfaces, the SALSA protein is mainly found associated with the epithelium and secreted into the surrounding fluids. The known features and functions of SALSA are presented in four panels (I–IV). (I) SALSA is present on the epithelial cell surface and deposited in the extracellular matrix, where it is involved in maintaining epithelial homeostasis. (II) Fluid-phase SALSA binds a broad array of microbes. It has been shown to agglutinate viruses, as well as both Gram-positive and Gram-negative bacteria thus preventing them from invading the parenteral spaces. (III) SALSA interacts with other endogenous molecules present at the mucosal surfaces, such as surfactant proteins SpA and SpD as well as IgA. It is believed that these molecules cooperate in antimicrobial defense. (IV) In the case of epithelial damage, cells and molecules from the tissue become mixed with the luminal contents. In this context, SALSA may bind the complement sensor molecules C1q, MBL, and the ficolins, thereby SALSA could initiate complement activation against distinct microbes or participate in the clearance of injured tissue components. **(B)** In its molecular structure, SALSA contains a stretch of 13 scavenger receptor cysteine-rich (SRCR) domains separated by SRCR interspersed domains. These are followed by two C1r/C1s, urchin embryonic growth factor and bone morphogenetic protein-1 (CUB) domains surrounding the 14th SRCR domain. Finally, a zona pellucida (ZP) domain is found at the most C-terminal end of the protein.

On the mucosal surfaces, SALSA has been shown to regulate the local immune system. On one hand, it scavenges invading microorganisms, whereas, on the other hand, it interacts with the mucosal epithelium to improve the integrity of this physical barrier (Figure 1A) (13, 39). SALSA binds a broad range of microbes, including viruses and bacteria (Table 1). Studies have shown that SALSA in the oral and intestinal mucosal secretions is sufficient to suppress infection by agglutinating microorganisms and keeping them from infecting the tissue. This has been observed for *Salmonella enterica*, HIV-1, and influenza A-virus (IAV) (27, 29, 30, 40). These studies suggested that SALSA simply functioned by agglutinating the microbes. However, the role of SALSA appears to be more complex than that. SALSA binds, e.g., to epithelial and tooth surfaces in addition to being secreted into the fluid phase (23). The epithelium-attached localization of a protein with a solely bacteria-agglutinating function would not appear to be beneficial for the human host. This paradox has been made clear by studies showing that SALSA, in some cases, may actually be exploited by the invading microbes. A study of dental caries showed that certain variants of the SALSA protein correlated positively with *Streptococcus mutans* adhesion to SALSA-coated hydroxyapatite surfaces and the development of dental caries. Other SALSA variants displayed the opposite correlation (41). In the case of HIV-1 infection, the salivary fluid SALSA protein was found to interfere with oral transmission. However, SALSA expressed on the vaginal epithelium had an enhancing effect on the infectivity of the virus (35). These findings suggest that some microbes have evolved mechanisms to utilize SALSA to infect the human body.

### Isoforms of the SALSA Protein

As indicated above, various variants of SALSA may interact differently with microbes. Indeed, different SALSA isoforms have been identified on various mucosal surfaces. These have been shown to vary both in protein sequence and in the glycosylation patterns (23, 36, 37). The gene for SALSA (in chromosome 10q26.13) encodes 13 highly conserved scavenger receptor cysteine-rich (SRCR) domains. These 109-amino acid-long motifs are found as “pearls on a string” separated by SRCR interspersed domains (SIDs) (Figure 1B). The stretch of 13 SRCR domains is followed by 2 C1r/C1s, urchin embryonic growth factor and bone morphogenetic protein-1 (CUB) domains encompassing the 14th SRCR domain. Finally, a zona pellucida (ZP) domain is found at the most C-terminal end (31, 42). The repetitive sequence of SRCR domains may facilitate alternative splicing (43). Indeed, mRNA transcripts encoding between 8 and 13 of the N-terminal SRCR domains have been observed, all in all revealing up to 7 distinct alleles (31, 42, 44). It has been estimated that SALSA contains 25–45% (w/w) of carbohydrate (8, 31). SALSA contains all the major sugar components. However, differences have been found to correlate to the secretor [Se(+/−)] status (±expression of the α1-2fucosyl-transferase). The blood group antigens, ABO, and Lewis antigens b and y (Le^b^ and Le^y^) were found on SALSA from Se(+) individuals. In contrast, SALSA from Se(−) individuals did not contain ABO, Le^b^ nor Le^y^ antigens. Instead, Lewis antigens a and x (Le^a^ and Le^x^) were present (45, 46). Thus, different forms of the SALSA protein exist. They are produced both by variations in the protein chain and in the extent and nature of glycosylation. The SALSA protein composition varies not only between individuals but also in different body compartments within the same individual (23, 36, 37).

## Salsa and Complement

### Interactions of C1q, MBL, and Ficolins with SALSA

C1q, MBL, and ficolins all form bouquet-like structures, where each subunit contains a collagen-like domain (stalk) and a carboxy-terminal globular domain (the “flower”) (47, 48). C1q binds specifically to surface-attached IgG and IgM. However, other endogenous non-immunoglobulin ligands have been found, including SALSA (4, 6). Also, MBL, M-ficolin, H-ficolin, and L-ficolin were found to bind to SALSA (6). All interactions between SALSA and the complement molecules were calcium dependent.

C1q was shown to bind SALSA through the globular domain in a region close to the immunoglobulin-binding site (49). Similarly, it appears that MBL utilizes the globular carbohydrate recognition domain (CRD) for the interaction with SALSA. Due to the heavy glycosylation of SALSA, sugar structures may function as a target for the CRD of MBL (8, 31). When the binding of MBL was tested to SALSA purified from the saliva of a single donor up to 60% inhibition of the SALSA–MBL interaction was observed when 5 mM fucose was added to the fluid phase (7). MBL binds to the Le^b^ antigen, a fucose-containing oligosaccharide (7). A clear difference was observed in the binding of MBL to SALSA from secretors vs. non-secretors (7). This correlates to the finding that only SALSA from Se(+) individuals contains the Le^b^ antigen (45, 46). This strongly suggests that MBL binds *via* the CRD to the Le^b^ antigen of SALSA.

### Complement Activation and Regulation by SALSA

It has been shown that the binding of C1q to SALSA is sufficient to initiate activation of the classical complement activation pathway (4, 6). In addition, SALSA was shown to influence the activation of the lectin pathway through interactions with MBL and the ficolins (5, 6). The overall outcome of SALSA-mediated complement regulation varies with the specific location of SALSA (6). SALSA coated onto a microtiter plate surface activated complement as measured by deposition of C4b and C3b after incubation with normal human serum (NHS). Using MBL-deficient serum, approximately 30% of the total complement activation was lost (6). The residual activation is likely mediated by C1q and possibly also by the ficolins (4, 6). In contrast to complement activation observed by surface-bound SALSA, SALSA in the fluid phase caused a dose-dependent inhibition of the lectin pathway (6). No such effect was observed on the classical pathway, which may be due to the almost 100 times higher concentrations of C1q vs. MBL. SALSA was able to interfere with the binding of the MBL–MASP2 complex to surface-coated mannan. *Candida albicans* is a known target for MBL-mediated complement activation. When NHS was mixed with increasing concentrations of SALSA and incubated with *C. albicans* a dose-dependent inhibition of the deposition of both C4b and C3b was observed on the *Candida* surface (6). The dual effects of SALSA on the complement system appear contradictory at first glance. On one hand, by binding to MBL and ficolins in the fluid phase, SALSA can prevent their binding to targets. On the other hand, when bound to a surface, SALSA can direct complement activation against appropriate targets, such as microbes. Overall, it appears that SALSA is a mucosal first line recognition molecule that can distinguish between targets to be cleared vs. structures to be tolerated.

Increased SALSA expression may alone and in concert with, e.g., C1q and MBL, lead to increased microbial clearance. In addition to the interactions with the complement proteins, SALSA can also mediate its anti-bacterial and inflammation regulating functions through interactions with IgA, SpA, and SpD (8, 9, 16). The functional outcome of these interactions is a cooperative effect on the microbial agglutination (Figure 1A) (50, 51). SALSA, SpA, and SpD have a dual effect against IAV: viral agglutination and inflammatory modulation (52). The binding of the SALSA ligand SpD to IAV has been shown to induce a strong respiratory burst response in neutrophils *in vitro*. This response was reduced by the addition of SALSA (51). It has been suggested that this allows a regulated response by the neutrophils, with an increased uptake of IAV but without an excessive and potentially harmful burst response (13). A similar feature is observed in the case of C-reactive protein and the other pentraxins. They target C1q to apoptotic and necrotic tissue, while simultaneously recruiting factor H to limit the complement activation (53, 54). This process is relevant during the removal of apoptotic debris at the mucosal surfaces, as well (55). The differential outcome of the interaction of SALSA with complement may represent a similar balanced effector mechanism against invading microbes.

## Salsa and Complement in Inflammatory Bowel Disease

Intestines are one of the primary sites, where an imbalance between activation and control of immune responses leads to disease. Inflammatory bowel disease (IBD) encompasses two chronic relapsing and remitting inflammatory conditions of the gastrointestinal tract. These are known as ulcerative colitis (UC) and Crohn’s disease (CD). Together, they affect up to 1:250 in the adult population (56). In children, the incidence of IBD is on the rise. Disease onset is during childhood or adolescence for up to 25% of the patients; although the mortality of the disease has been declining, it has a major impact on the development of these young individuals (57). The fundamental causes of the diseases are still obscure.

Several associations have been found between complement components and IBD. Specifically for the gut mucosa, the development of CD has been associated with an altered expression of components of the lectin pathway. The frequency of the *MBL2* gene allele, which results in MBL deficiency, was significantly elevated in pediatric patients with CD compared to healthy controls or adults with Sjögren’s syndrome (58, 59). Deficiencies in classical and alternative pathway components are rarer. Some patients deficient in C1 inhibitor, which is commonly associated with hereditary angioedema, were found to develop non-infectious enteritits and IBD (60–62). To further highlight an involvement of the classical and lectin pathways of complement, we recently observed an association of pediatric IBD to an MHC haplotype that involves a deficiency of two C4 genes (HLA-A03; HLA-B07; one C4A gene; one C4B gene; HLA-DRB115) (63).

A study of lectin pathway components during CD treatment found a dramatic impact on M-ficolin and MASP-3 levels in patients responding to anti-TNF-α therapy (64). However, how the complement components specifically affect the local inflammatory environment of the gut is not clear yet. The above described interactions with the SALSA molecule present a potential way for complement to affect a balanced mucosal immunological response. Current models of CD pathogenesis include an altered response to the local microbiota, and an increased SALSA expression has been linked to several of these responses (65). Studies have shown that SALSA can be strongly induced by various immunological stimuli (66, 67). The increased levels of SALSA in the intestinal epithelium of patients with IBD and in the ethmoid sinusoidal mucosa of patients with chronic sinusitis suggest that SALSA expression is part of the mucosal inflammatory response (66–68). Furthermore, a study of preterm infants revealed that an increase in the pulmonary SALSA levels was part of the mucosal response to neonatal infection (69).

Salivary scavenger and agglutinin expression by the intestinal epithelium is induced by NOD2 and TLR4 activation (27). However, the outcome of an induced SALSA expression during IBD may not necessarily lead to enhanced clearance only. Rather, the interaction of SALSA with several endogenous molecules may be part of an efficient but limited immunological response. Failure in these processes could propagate an unbalanced and overactive local immune response in IBD. It has been shown that the previously described SALSA isoforms influence both the interaction with microbes and the endogenous ligands, such as IgA, C1q, and MBL (37). Interestingly, the specific bacterial-binding ability of SALSA has been found to depend not only on the isoform of the protein but also on the location of the protein. Fluid-phase SALSA can bind and aggregate some streptococcal strains, while SALSA coated to a hydroxyapatite surface does not (23). Thus, the association with the mucosal epithelium or the secretion into the lining fluids may further affect the local immunological environment differently. Finally, the described interaction of SALSA and trefoil factors, being important in maintaining the mucosal epithelial barrier, has also been suggested to play a role in IBD (70, 71).

Salivary scavenger and agglutinin may be part of the normal immunological response of the mucosal epithelium during infection. Individual variations in the expression of SALSA isoforms alternate the ability of SALSA to interact with endogenous ligands, to invade microbes, and perhaps even to induce a limited burst response in neutrophils. It is therefore not surprising that a specific SALSA isoform, lacking the five most N-terminal SRCR domains, has been associated with CD (65, 67).

We speculate that the individual variations in the SALSA interactions are key in understanding how this molecule could play a role in shifting the immunological balance toward increased inflammation at the mucosal surfaces, with detrimental effects for IBD patients.

## Future Perspectives

At the mucosal surfaces, a very tight immunological response to infection and inflammation is essential. The SALSA molecule is central player interacting with a multitude of endogenous molecules, invading microbes and the epithelial barrier. Due to the tightly linked interactions, a balanced function of the SALSA molecule is key in avoiding an overactive immune response. The interplay between the various SALSA isoforms, ficolins, MBL, and C1q with modified tissue components, carbohydrates, acetylated molecules, and microbes on mucosal surfaces provides an interesting area for future research that may open a new understanding of mechanisms underlying the development of mucosal immunological disorders.

## Author Contributions

Both authors contributed to the design and writing of this review.

## Conflict of Interest Statement

The authors declare that the research was conducted in the absence of any commercial or financial relationships that could be construed as a potential conflict of interest.
